# An Experimental Investigation of the Degradation of CMOS Low-Noise Amplifier Specifications at Different Temperatures

**DOI:** 10.3390/mi13081268

**Published:** 2022-08-06

**Authors:** Shaohua Zhou, Jian Wang

**Affiliations:** 1 School of Microelectronics, Tianjin University, Tianjin 300072, China; 2 Qingdao Institute for Ocean Technology, Tianjin University, Qingdao 266200, China; 3 Shandong Engineering Technology Research Center of Marine Information Perception and Transmission, Qingdao 266200, China

**Keywords:** CMOS, specification degradation, temperature, LNA

## Abstract

To investigate the relationship between the specifications degradation of a low-noise amplifier (LNA) and temperature, we experimentally investigated the degradation characteristics of the specifications of the LNA at different temperatures. The small-signal gain (S21) of the LNA decreases with increasing temperature. This paper discusses and analyzes the experimental results in detail, and the reasons for the degradation of LNA specifications with temperature changes are known. Finally, we have tried to use the structure already available in the literature for the PA temperature compensation circuit for the temperature compensation of the LNA. The results show that the existing circuit structure for PA temperature compensation in the literature can also effectively compensate for the S21 and NF degradation of the LNA due to the temperature increase.

## 1. Introduction

A low-noise amplifier (LNA) is used in the first stage of a radio frequency (RF) transceiver to amplify a signal with low noise [1]. At the same time, the specification of the LNA will change with the change in temperature because the parameters of semiconductors are very sensitive to the change in temperature [2,3]. Therefore, their system will fail when the specifications of LNAs decrease with the temperature change; thus, they cannot meet the minimum requirements required for the system’s normal operation.

Current research on LNAs focuses on the optimization of performance specifications, such as bandwidth [4,5,6], linearity [7,8,9], gain [10,11], noise figure [12,13], and power consumption [14,15,16]. For example, in 2016, G. Nikandish et al. [4] proposed a feedback amplifier circuit to increase the bandwidth of the LNA. In 2016, Sunhwan Jang et al. from the United States published a paper entitled “A High-Gain Power-Efficient Wideband V-Band LNA in 0.18 μm SiGe Bipolar Complementary Metal-Oxide Semiconductor (BiCMOS)”, in which Sunhwan Jang et al. used an effective wideband gain shaping method to improve the gain of LNAs at low power consumption [11]. Furthermore, at the 2018 18th Mediterranean Microwave Symposium, O. Memioglu et al., scholars from Middle East Technical University, presented a cascaded topology with excellent noise figures consisting of cascaded topology with inductive source degradation of co-sourced devices [7]. In 2020, Yuito Sawayama et al. from Okayama Prefectural University in Japan achieved a low-noise figure LNA by replacing the primary inductor with an external inductor in the input matching circuit [13]. In 2021, Roman Yu. Musenov, an academic from Russia, et al. proposed a single-ended low-power LNA based on 90 nm CMOS technology, which achieves low power consumption by employing current reuse techniques [15]. These studies focus on improving the bandwidth, linearity, and gain of LNAs, and reducing the noise figure and power consumption.

In this paper, we investigate the relationship and law of degradation of LNA specifications with temperature changes, taking 50 MHz–450 MHz CMOS LNAs as an example. The reasons for the degradation of LNA specifications, such as gain and noise figure with increasing temperature, are discussed and analyzed. Based on this, we also designed a circuit for compensating the temperature characteristics of this LNA, which consists of a diode and two resistors. The simulation results show that this compensation circuit can effectively compensate for the degradation of S21 and NF due to temperature. The results can provide a theoretical reference for the design of the temperature compensation circuit of LNA. On the other hand, it can provide effective guidance for optimizing the system design.

## 2. Structure and Experimental Setup of LNA

### 2.1. The Structure of LNA

**Figure 1:**
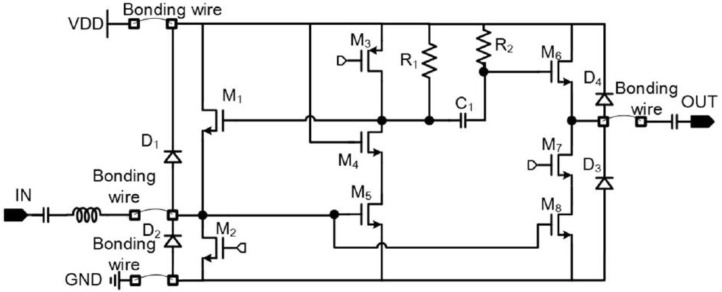
The schematic diagram of the LNA [17].

**Figure 2:**
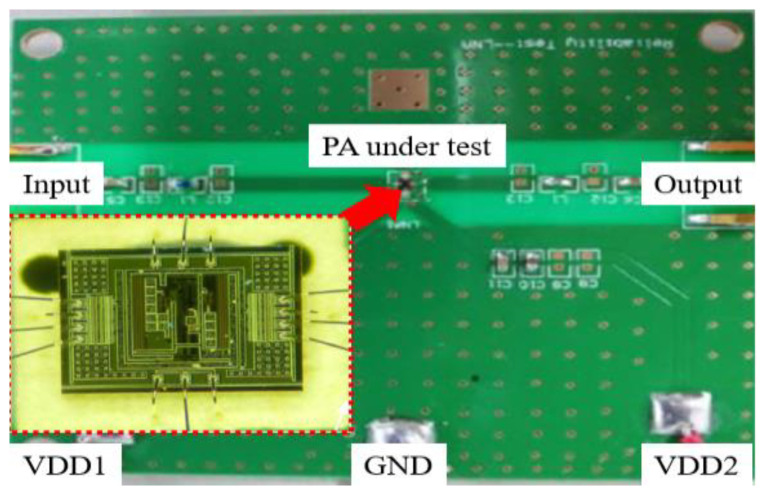
The micrograph of the chip of the LNA.

The schematic diagram of the LNA and the chip micrograph are shown in Figure 1 and Figure 2, respectively.

As shown in Figure 1, the LNA is manufactured using a 0.18 um CMOS process [17]. In this LNA, a shunt feedback structure is used in the matching stage to reduce the noise factor of the LNA [17]. The load of the matching stage uses a PMOS (positive channel metal oxide semiconductor) current source in parallel with a resistor, mainly to relax voltage headroom and achieve adequate performance in process corners [17,18].

Figure 2 shows a micrograph of the chip of the LNA, which was gold wire bonded to a PCB (printed circuit boards) board to facilitate temperature characterization experiments in an environmental chamber. The left and right ends of the PCB are the input and output, respectively, which are connected to the test system via a 50-ohm matched subminiature version A (SMA). In addition, external inductors and resistors are used at the input and output of the PCB to achieve better matching. Two supply voltages are used for this LNA, 1.8 V and 3.3 V, respectively.

### 2.2. Experimental Environment and Setup

**Figure 3:**
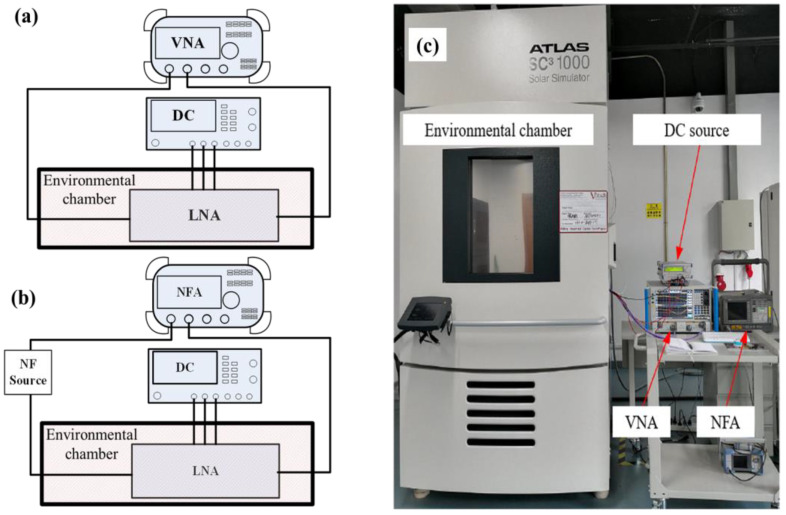
Measurement environment and setup: (**a**) a schematic diagram of the S-parameter measurement; (**b**) a schematic diagram of the NF measurement; (**c**) a physical diagram of the measurement environment.

As shown in Figure 3, the experiments were conducted in a chamber (SC^3^ 1000) that can support conducting high- and low-temperature experiments from −40 °C to 125 °C. However, due to the constraints of the experimental cable, only the temperature characteristics of the LNA from −40 °C to 90 °C are investigated.

In the experimental process, we focused on the temperature characteristics of the gain and noise figure. The gain and noise figures were measured with a vector network analyzer (VNA) (AV3672B) and a noise figure analyzer (N8975A). In addition, a Rohde & Schwarz power supply (NGMO2) was used for the power supply required in the experiments.

## 3. Results and Discussions

### 3.1. The S21

**Figure 4:**
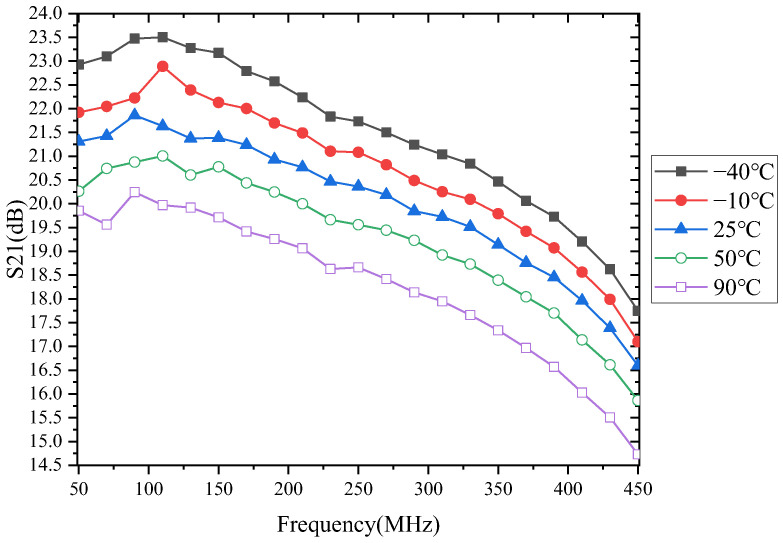
Measured gain with frequency variations.

The temperature characteristics of the LNA gain are shown in Figure 4. From the figure, we can see that S21 is not only a function of temperature, but also a function of frequency. As the temperature increases, the S21 of the LNA gradually decreases. S21 decreases with increasing frequency, only differing at individual points. This may be caused by jitter in the measurement system during the measurement process.

The specific causes of the degradation of the gain of the LNA with increasing temperature are discussed below. 

The expressions for *I_DS_* in the linear and nonlinear regions, respectively, are [19,20]

(1) $$I_{DS-linear}=\frac{W\mu_{n}C_{ox}}{2L}\left[2(V_{GS}-V_{TH})V_{DS}-V_{DS}{}^{2}\right]$$

(2) $$I_{DS-nonlinear}=\frac{W\mu_{n}C_{ox}}{2L}{\left(V_{GS}-V_{TH}\right)}^{2}$$

where *W* is the gate width, *μ_n_* is the carrier mobility, *C_ox_* is the gate oxide capacitance per unit area, *L* is the gate length, *V_GS_* is the gate voltage, *V_TH_* is the threshold voltage, and *V_DS_* is the drain voltage.

According to Equations (1) and (2), the transduction in the linear and nonlinear regions is [19,20]

(3) $$g_{m-linear}=\sqrt{2\mu_{n}\frac{WC_{ox}}{L}I_{DS-linear}}$$

(4) $$g_{m-nonlinear}=\sqrt{2\mu_{n}\frac{WC_{ox}}{L}I_{DS-nonlinear}}$$

where the expression for the carrier mobility is [21]

(5) $$\mu_{n}(T)=\mu_{n}(T_{0}){\left(\frac{T}{T_{0}}\right)}^{-m}$$

where *T*_0_ = 300 K, *m* = 1.5~2.

According to Equation (5), the *μ_n_* degrades with increasing temperature. And according to Equations (3) and (4), it is known that the transconductance of both linear and nonlinear regions is positively related to carrier mobility. Therefore, the transconductance of both linear and nonlinear regions will degrade with increasing temperature. In addition, the transconductance is the gain [19,20]; thus, the LNA’s gain is also degraded as the temperature increases. Therefore, the decrease in S21 with increasing temperature decreases carrier mobility with increasing temperature.

**Figure 5:**
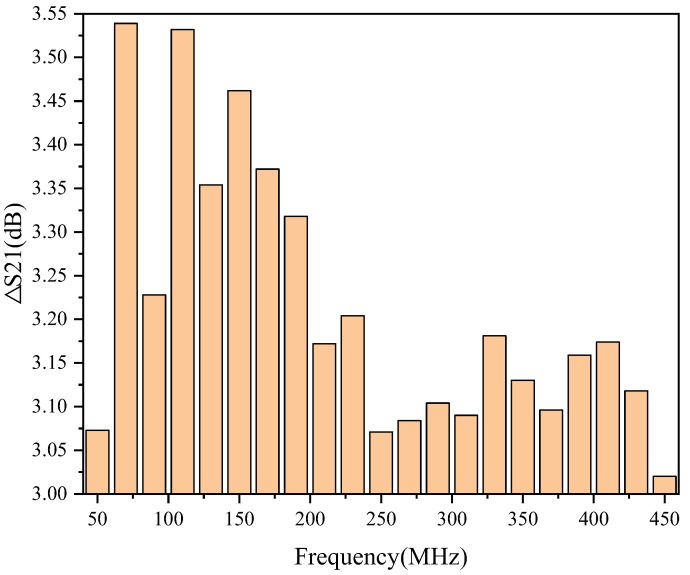
Variation of S21 in the temperature range from −40 °C to 90 °C.

We already know that the S21 degrades as the temperature rises. Figure 4 shows that when the temperature change range is the same, the S21 changes at different frequencies are also different. For example, Figure 5 shows the evolution of S21 in the temperature range of −40 °C to 90 °C.

As shown in Figure 5, S21 decreases by 3.54 dB, 3.2 dB, and 3.02 dB for 70 MHz, 230 MHz, and 450 MHz, respectively, when the temperature increases by 130 °C. This indicates that the *μ_n_* is temperature-dependent and frequency-dependent.

### 3.2. The Noise Figure (NF)

**Figure 6:**
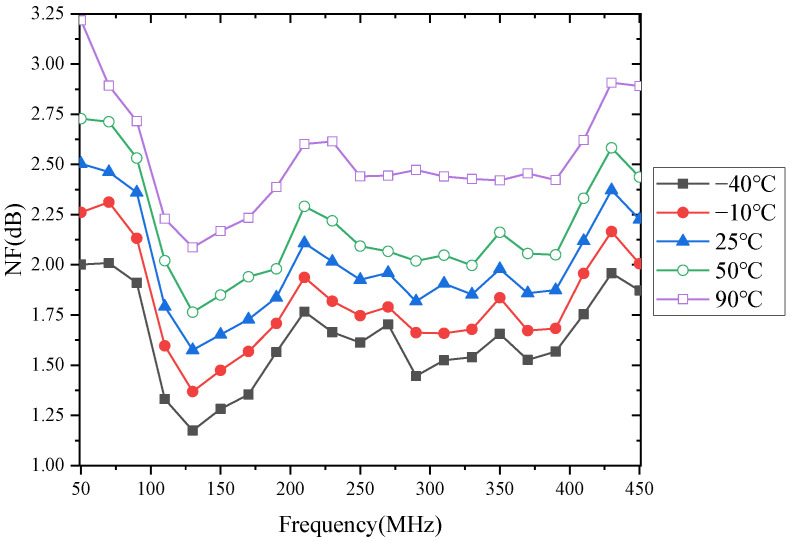
Measured noise figure with frequency variations.

The noise figure of the LNA as a function of temperature is shown in Figure 6. As shown in Figure 6, the noise coefficient of the LNA increases with temperature; this means that the noise performance of the LNA degrades with increasing temperature. In addition, the noise figure of the LNA varies with frequency rather than monotonically. That is, the noise figure of the LNA increases or decreases with increasing frequency. This indicates that the noise figure of the LNA is not only a function of temperature, but also a function of frequency.

The following is a specific discussion of the causes of the increase in the noise coefficient of the LNA with increasing temperature. The expression of the *NF_min_* of LNA is [22]

(6) $$NF_{\min}=1+2\pi K(C_{gs}+C_{gd})\sqrt{\frac{R_{s}+R_{d}}{g_{m}}}$$

where *K* is the Fukui constant, *C_gs_* and *C_gd_* are the gate-source and gate-drain capacitance, *R_s_* and *R_d_* are the source and drain resistance, and *g_m_* is the transconductance.

The transconductance degrades with increasing temperature. In addition, the source/drain resistance also increases with temperature. Therefore, according to Equation (6), the increase in source/drain resistance and the degradation of the transconductance will increase the NF_min_ of the LNA as the temperature increases. Consequently, there is an increase in the LNA noise figure with the temperature rise. This indicates that the increase in source/drain resistance and the transconductance degradation are the two main reasons for the rise in the noise figure with increasing temperature.

**Figure 7:**
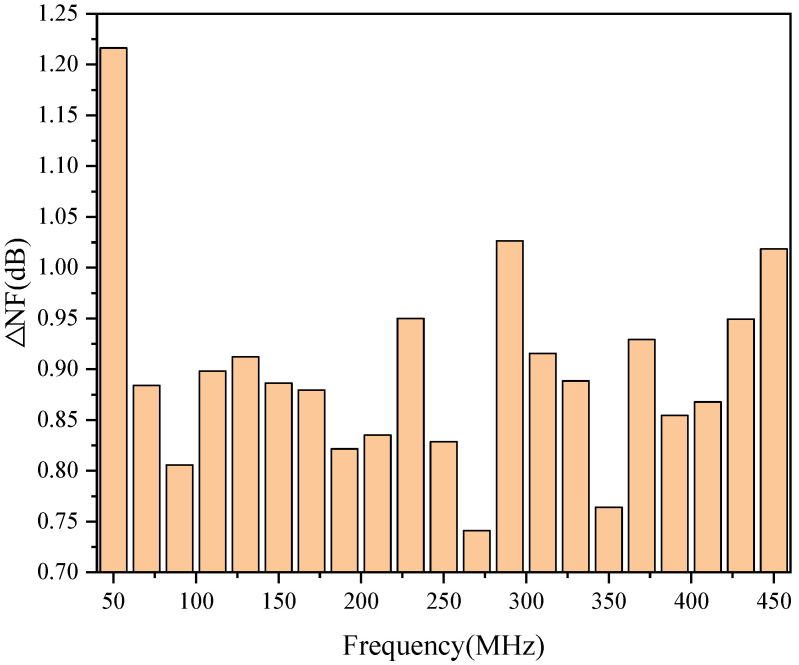
Variation of the NF in the temperature range from −40 °C to 90 °C.

It is already known from Figure 6 that the noise figure of the LNA increases with temperature. This is because the NF changes differently for different frequencies in the same temperature variation range. For example, Figure 7 shows the variation of the noise figure to varying frequencies in the field of −40 °C to 90 °C. As shown in Figure 7, the noise figure at 50 MHz, 270 MHz, and 450 MHz increases by 1.22 dB, 0.74 dB, and 1.02 dB, respectively, when the temperature increases from −40 °C to 90 °C. This also shows that the *μ_n_* is not only temperature-dependent but also frequency-dependent.

## 4. On-Chip Temperature Compensation for LNAs

**Figure 8:**
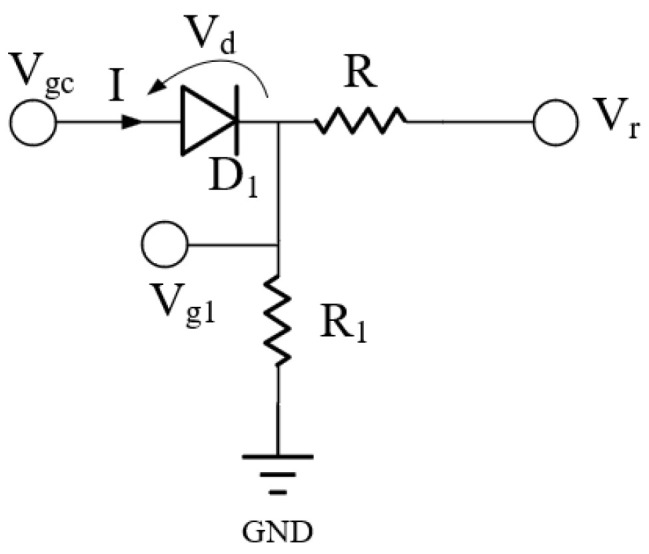
Temperature compensation circuit structure for the LNA [23].

A circuit structure for multistage PA temperature compensation was proposed in the literature [23]; based on this structure, we propose a temperature compensation circuit for stacked PAs [24]. Considering the simple structure of this compensation circuit and our existing research base, in this subsection, we try to use the temperature compensation circuit structure for the multistage PA proposed in the literature [23] for the compensation of S21 and NF of the LNA. Furthermore, we explore whether the compensation circuit structure presented in [23] can be used for the temperature compensation of other microwave/RF circuits such as LNAs in addition to multistage PAs. The structure of the circuit used for LNA temperature compensation in this subsection is the same as that used for the multistage PA temperature compensation circuit in the literature [23], as shown in Figure 8. As shown in Figure 8, this compensation circuit consists of a diode (D_1_) and two resistors (R and R_1_).

Since the circuit structure used is the same as that of the literature [23], the temperature compensation principle of this temperature compensation circuit for the LNA is also the same as that of the literature [23]. Therefore, to avoid repetition, it is not presented again.

According to the literature [23], when the temperature increases from *T_L_* to *T_H_*, Equations (1) and (2) can be expressed, respectively, as

(7) $$I_{DS-linear}=\frac{W\mu_{n}C_{ox}}{2L}\left[2((V_{GS}+\Delta V_{g1})-V_{TH})V_{DS}-V_{DS}{}^{2}\right]$$

(8) $$I_{DS-nonlinear}=\frac{W\mu_{n}C_{ox}}{2L}{\left((V_{GS}+\Delta V_{g1})-V_{TH}\right)}^{2}$$

where Δ*V_g_*_1_ is the variation of *V_g_*_1_ between two temperatures (*T_H_* and *T_L_*).

According to Equations (7) and (8), the *I_DS_* will increase with the increase of Δ*V_g_*_1_. Furthermore, from Equations (3) and (4), the transconductance is proportional to the drain current; i.e., it increases with the increase of the *I_DS_*. This means that the temperature compensation circuit increases the transconductance with the rise in the Δ*V_g_*_1_; thus, the temperature compensation of the transconductance is realized. That is, the compensation of S21 is realized.

**Figure 9:**
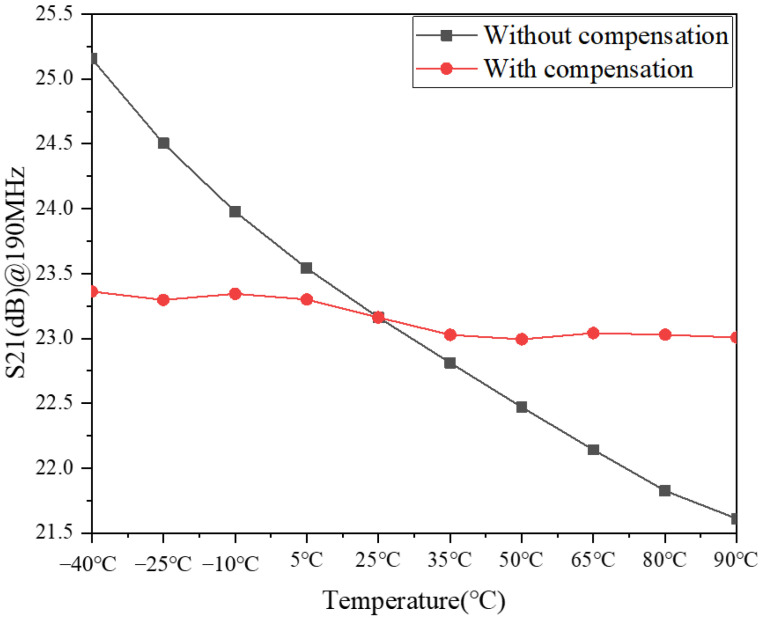
Simulation results of S21 with and without compensation.

Figure 9 gives the simulation results of S21 with and without compensation, from which when the temperature increases from −40 °C to 90 °C, S21 changes by 0.4 dB with compensation, while S21 changes by 3.5 dB without compensation; this indicates that the compensation circuit is effective in compensating S21 with a temperature change.

**Figure 10:**
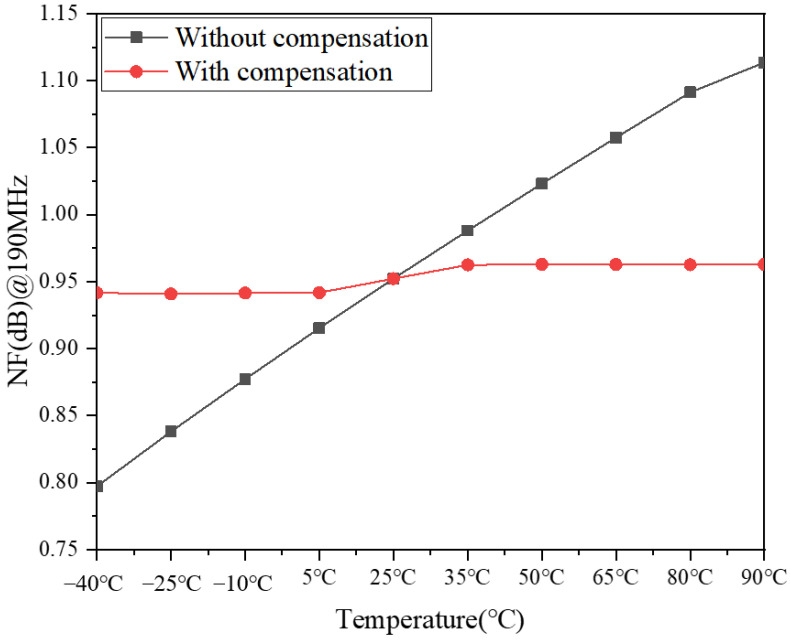
Simulation results of the NF with and without compensation.

From Equation (6), NF will decrease with the increase of the transconductance. Therefore, under the temperature compensation circuit, NF will reduce with the rise of the Δ*V_g_*_1_; thus, this will achieve the temperature compensation of NF. The simulation results for the NF with and without compensation are shown in Figure 10. It can be seen from this that the NF with temperature compensation varies less with temperature, while the NF without temperature compensation increases with temperature. Specifically, when the temperature increases from −40 °C to 90 °C, the NF changes by 0.02 dB with temperature compensation, while the NF changes by 0.3 dB without temperature compensation. This indicates that the temperature compensation circuit effectively compensates for the NF degradation with temperature increase.

The above is the working principle of the temperature compensation circuit proposed in this subsection, mainly to increase the transconductance by increasing the gate voltage to achieve the compensation of S21 and NF with temperature change. The simulation results in Figure 9 and Figure 10 show that the temperature compensation circuit structure proposed in the literature [23] applies not only to the temperature compensation of PA, but also to the temperature compensation of LNA.

## 5. Conclusions

This paper investigates the key specifications of LNAs, such as gain and noise figures, from an experimental point of view at different temperatures using a CMOS 50 MHz–450 MHz LNA as an example. The S21 decreases with temperature increase while the noise figure increases. Furthermore, it is found that one of the leading causes of the degradation of the gain and noise characteristics is the degradation of the transconductance. The results can guide temperature-compensated circuit design to complement the degradation of the specification. On the other hand, it is also proved that the temperature compensation circuit structure proposed in the literature [23] applies to the temperature compensation of PAs and can be used for the temperature compensation of LNAs.

The specification degradation of the LNA is not only related to temperature, but also to humidity, high- and low-temperature shock, and time. Therefore, future work in this paper will consider the relationship between the specification degradation of the LNA with humidity, the number of high- and low-temperature shocks, and time.

## Data Availability

Not applicable.
